# Homeopathic and conventional treatment for acute respiratory and ear complaints: A comparative study on outcome in the primary care setting

**DOI:** 10.1186/1472-6882-7-7

**Published:** 2007-03-02

**Authors:** Max Haidvogl, David S Riley, Marianne Heger, Sara Brien, Miek Jong, Michael Fischer, George T Lewith, Gerard Jansen, André E Thurneysen

**Affiliations:** 1 Ludwig Boltzmann Institute for Homeopathy, Graz, Austria; 2 University of New Mexico School of Medicine and Integrative Medicine Institute, Santa Fe, New Mexico, USA; 3 HomInt, Karlsruhe, Germany; 4 Complementary Medicine Research Unit; Primary Medical Care, University of Southhampton, Southhampton, UK; 5 VSM Geneesmiddelen, Alkmaar, The Netherlands; 6 ClinResearch GmbH, Cologne, Germany; 7 Tilburg, The Netherlands; 8 Institute for Complementary Medicine (KIKOM), University of Bern, Bern, Switzerland; 9 passed away in 2005

## Abstract

**Background:**

The aim of this study was to assess the effectiveness of homeopathy compared to conventional treatment in acute respiratory and ear complaints in a primary care setting.

**Methods:**

The study was designed as an international, multi-centre, comparative cohort study of non-randomised design. Patients, presenting themselves with at least one chief complaint: acute (≤ 7 days) runny nose, sore throat, ear pain, sinus pain or cough, were recruited at 57 primary care practices in Austria (8), Germany (8), the Netherlands (7), Russia (6), Spain (6), Ukraine (4), United Kingdom (10) and the USA (8) and given either homeopathic or conventional treatment. Therapy outcome was measured by using the response rate, defined as the proportion of patients experiencing 'complete recovery' or 'major improvement' in each treatment group. The primary outcome criterion was the response rate after 14 days of therapy.

**Results:**

Data of 1,577 patients were evaluated in the full analysis set of which 857 received homeopathic (H) and 720 conventional (C) treatment. The majority of patients in both groups reported their outcome after 14 days of treatment as complete recovery or major improvement (H: 86.9%; C: 86.0%; *p *= 0.0003 for non-inferiority testing). In the per-protocol set (H: 576 and C: 540 patients) similar results were obtained (H: 87.7%; C: 86.9%; *p *= 0.0019). Further subgroup analysis of the full analysis set showed no differences of response rates after 14 days in children (H: 88.5%; C: 84.5%) and adults (H: 85.6%; C: 86.6%). The unadjusted odds ratio (OR) of the primary outcome criterion was 1.40 (0.89–2.22) in children and 0.92 (0.63–1.34) in adults. Adjustments for demographic differences at baseline did not significantly alter the OR. The response rates after 7 and 28 days also showed no significant differences between both treatment groups. However, onset of improvement within the first 7 days after treatment was significantly faster upon homeopathic treatment both in children (*p *= 0.0488) and adults (*p *= 0.0001). Adverse drug reactions occurred more frequently in adults of the conventional group than in the homeopathic group (C: 7.6%; H: 3.1%, *p *= 0.0032), whereas in children the occurrence of adverse drug reactions was not significantly different (H: 2.0%; C: 2.4%, *p *= 0.7838).

**Conclusion:**

In primary care, homeopathic treatment for acute respiratory and ear complaints was not inferior to conventional treatment.

## Background

The evidence base for complementary and alternative medicine (CAM) in general is limited and there is certainly a need for more research in areas such as homeopathy [1]. Objective data collection and evaluation is needed to assist physicians in patient care and advance the quality of medical practice [2]. Clinical trials, especially randomised controlled trials (RCTs), are generally accepted as producing the highest level of evidence for medical interventions. Driven by the discovery of new pharmaceutical substances, demands from regulatory authorities for clinical data and the need of physicians for evidence based treatment strategies, the methodology of RCTs became the subject of research itself. Within this context, the strengths and weaknesses of such trials have been debated [3]. Placebo-controlled RCTs are indispensable for the development of pharmaceutical agents with unknown efficacy and safety profiles. Their limitations result from highly standardized study protocols and patient populations, which may create artificial situations that differ from daily practice. Moreover, even the fact that patients are enrolled into a placebo-controlled clinical trial will influence treatment outcome, sometimes leading to high placebo or low verum response rates [4]. Consequently, more practice-based studies have been developed such as pragmatic RCT's or non-randomised cohort studies. Especially non-interventional outcomes studies have only few inclusion and exclusion criteria. Therefore they may provide information about a broad and heterogenous patient population thus resulting in high external validity for daily medical practice. However, the fact that patients are not randomly assigned to treatments in such outcome studies may lead to baseline differences between groups and makes the interpretation of the results more susceptible to bias. This disadvantage may be overcome, at least in part, by the application of statistical methods to control for baseline differences between treatment groups.

Apart from the ongoing discussion about clinical evidence, complementary therapies are well integrated into primary care in most Western countries. Among these, homeopathy is the most frequently used form in various acute and chronic conditions [5-9]. The value of homeopathy in chronic conditions has been demonstrated in several studies. A comprehensive analysis of outcome and cost-effectiveness showed that chronically ill patients had a better overall outcome with homeopathic than with conventional care [10]. Another large-scale observational study showed a positive impact of homeopathy on the health status in a substantial proportion of patients suffering from a wide range of different chronic diseases [11]. To our knowledge, no large comparative cohort studies have been performed to investigate the outcome of homeopathic treatment for acute illnesses. Results of the first phase of this study, the International Integrative Primary Care Outcomes Study 1 (IIPCOS-1), suggest that homeopathic treatment is at least as effective as conventional treatment for acute complaints of the upper and lower respiratory tract [12]. The aim of the present study, IIPCOS-2, was to evaluate on an international basis and in a large sample size if homeopathic treatment is non-inferior to conventional treatment in patients with acute respiratory and ear complaints.

## Methods

### Study design

IIPCOS-2 is an international, multi-centre, comparative cohort study of non-randomised design, which was conducted between October 1998 and April 2000. Patients suffering from acute respiratory and ear complaints were recruited by physicians in 57 primary care practices in Austria (8), Germany (8), the Netherlands (7), Russia (6), Spain (6), Ukraine (4), United Kingdom (10) and USA (8). The physicians belonged to 3 different groups: providing homeopathic treatment only (22), providing either homeopathic or conventional treatment (9), and providing conventional treatment only (12). The physicians, prescribing primarily homeopathic single remedies, had in addition to their conventional medical qualifications, graduated from a homeopathic training program and at least 5 years experience using homeopathy in their medical practice. The protocol was approved by the International Ethics Committee in Freiburg, Germany. The study was conducted in accordance with the declaration of Helsinki, Good Clinical Practice (GCP) guidelines and national legal requirements.

### Patients

Patients older than one month, presenting themselves with at least one of five chief complaints (runny nose, sore throat, ear pain, sinus pain or cough), and onset of symptoms not more than 7 days before, were eligible to participate. Each chief complaint comprised of 5 to 9 individual symptoms, which were rated by the physicians with scores from 0 – not present to 4 – very severe. The mean score for each chief complaint was used to measure severity at baseline. Patients meeting the inclusion criteria, respectively in case of children their parents/legal guardians, were informed by the physician about the nature of the study. Prior to enrolment into the trial each patient/parent had to provide written informed consent to participate. Exclusion criteria were among others severe mental impairment, severe chronic diseases such as spinal cord injuries and alcohol or drug abuse. At centres providing both therapies (mixed centres) the treatment was chosen by the physicians and/or following the patients' preference.

### Study protocol

During the initial patient contact the physician documented the onset of chief complaint, severity of symptoms, clinical diagnosis, concomitant medical problems and medication and primary treatment prescribed. Patients completed a questionnaire asking for demographic and health-related information. Additionally some general questions addressed the patients' willingness to pay, patient confidence in health care provider and therapy, treatment preference, willingness to be randomized (at mixed centres only) etc. The patient follow-up was carried out by telephone 7, 14 and 28 days after the initial contact. Independent external study collaborators performed the calls. According to the study protocol they were blinded for the patient's treatment. The following parameters were documented: severity of complaint-related symptoms, time until occurrence of first improvement, therapy outcome (assessed with *complete recovery*, *major improvement*, *slight improvement*, *no change *or *deterioration*), patient's satisfaction with the treatment (*very satisfied, satisfied, neutral, dissatisfied *or *very dissatisfied*) and general health condition. In case any adverse events had occurred, the physician was informed in order to collect more information and medically assess the case.

The response rates were defined as the proportion of patients assessing themselves as 'completely recovered' or 'major improved' after 7, 14 and 28 days of treatment. The main outcome criterion was the response rate after 14 days. Other outcome criteria were the response rates after 7 and 28 days, time to onset of first improvement (patients' assessments after how many days they had experienced a first improvement), patient satisfaction with treatment and health care provider and the occurrence of adverse events. Adverse events were coded by using the WHO-ART terminology.

A total of 72 selected homeopathic medications in potencies of 12C and higher (manufactured according to the German Homeopathic Pharmacopoeia), were given to the physicians as the basic set of study medication. Nevertheless, the physicians were free to prescribe any other remedy, any other potency or dosage form. Conventional treatment, registered in each participating country, was prescribed by the investigator and picked from a pharmacy.

### Data collection and monitoring

Data were collected with a validated remote data entry system that was accessed via the Internet. The physicians entered their data online into electronic case report forms. The remote data entry system checked each entry for completeness and consistency. It recorded all data values with date and time of entry as well as all changes in the database in an audit trail. Access to the database was protected by password identification. Each user had a unique password that was provided in a sealed envelope. After entering was completed, data were transferred via Internet to the data collection centre at the former Institute for Numerical Statistics (IFNS, acquired by Omnicare Inc. in 1999) in Cologne, Germany. Monitoring was performed adherent to GCP-guidelines by an independent clinical monitor. Monitoring visits took place at least twice in order to inspect the course of the trial and to carry out source data verification. A data review tool enabled the monitor to identify missing data values, data values deviating from the normal range and among other things, data needing source verification.

### Statistical methods

Data analysis was conducted by ClinResearch, Cologne, Germany, using the statistical software package SAS 9.1.3 under Windows XP Professional. The study was designed to confirm non-inferiority of the primary outcome criterion in the total patient population after homeopathic treatment in comparison to conventional treatment, using the one-sided equivalence test at the 2.5% significance level. The non-inferiority margin was defined by 5%-points. Subgroup analyses were performed on age groups (children: < 18 years; adults: ≥ 18 years) with respect to demographic data, response rates, patient satisfaction and other outcome criteria using the Chi-square test, Fisher's exact test and Wilcoxon's rank sum test. The treatment groups were checked for baseline comparability and logistic regression analysis was performed to control for baseline differences. The primary and secondary outcome criteria were analysed on the full-set population, comprising all patients who received at least one dose of investigational medication and having at least one follow-up contact. Missing data in case of patient withdrawals from the trial were replaced by applying the last observation carried forward (LOCF) principle. A secondary analysis was performed on the per-protocol set population, comprising all patients with follow-up data on day 14.

## Results

### Patients

**Figure 1:**
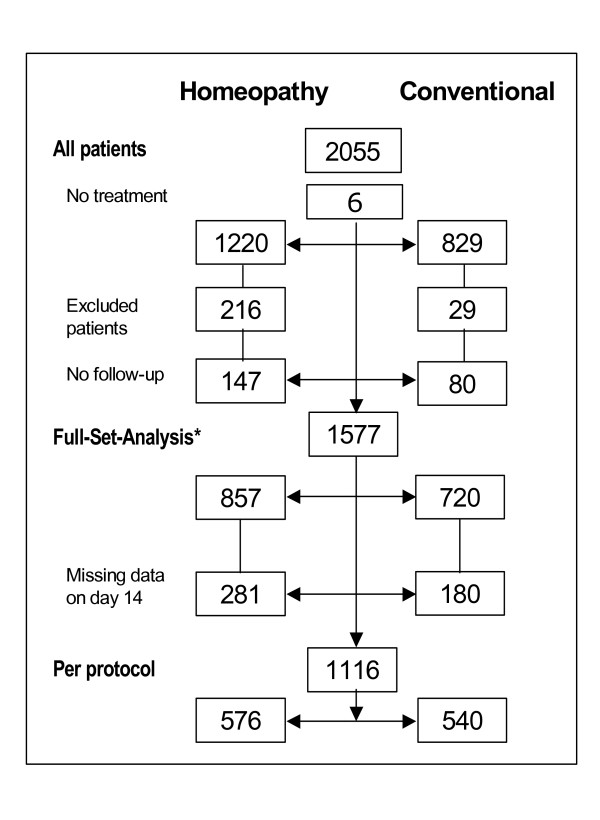
**Patient flow-chart**. * All patients who received at least one dose of investigational medication and having at least one follow-up contact.

A total of 2,055 patients suffering from at least one chief complaint (acute runny nose, sore throat, ear pain, sinus pain or cough) were enrolled in the study and given either homeopathic (H: n = 1,220) or conventional treatment (C: n = 829) (Figure 1). Six patients did not receive any treatment and were excluded from further analysis. All patients from the USA and Spain (H: n = 216; C: n = 29) were excluded since telephone interviews were not performed according to the study protocol. For another 227 patients no follow-up data were available because either interviews could not be carried out or the patient withdrew from the study. Data of 1577 patients with at least one follow-up contact were evaluated (full-set analysis), 857 patients in the homeopathy group and 720 patients in the conventional treatment group. For 1116 patients (H: n = 576; C: n = 540) follow-up data on day 14 were documented, being the per-protocol set (Figure 1).

Upon enrolment in the study, patients, or the patients' legal guardians were asked for their treatment preference. In the homeopathy group, 81% of patients had a preference for homeopathy, 18% had no treatment preference. In the conventional group, 55% of the patients' preferred conventional treatment, 2% homeopathy and 43% had no treatment preference. Patients at mixed centres were additionally asked whether they would agree to be randomized if the choice of treatment was made randomly. With 68.1%, the majority of patients in the homeopathy group refused to be randomized, 30.6% had no problem with randomisation and in 1.3% no remark was given. In the conventional group willingness and unwillingness to be randomized were equally distributed (51.9% yes, 47.9% no, 0.1% no remark).

### Baseline characteristics

Demographic data of children (< 18 years of age) and adults (≥ 18 years of age) are presented in Table 1. The proportion of children under 18 years was 47% of patients receiving homeopathic compared to 35% receiving conventional treatment. Within this subpopulation the average age and Body Mass Index (BMI) differed significantly between both treatment groups. In adults, the distribution of males and females, average age and BMI differed significantly between the homeopathic and conventional group.

**Table 1:** Demographic data

**Children**	**Homeopathy**, n = 407	**Conventional **n = 252	***p*-value, if < 0.05**
Male (%)	51.1	50.0	
Female (%)	48.9	50.0	
Age	6.6 ± 4.3	7.4 ± 4.7	= 0.0282^a^
BMI	16.6 ± 3.0	17.9 ± 3.7	= 0.0001^a^

**Adults**	**Homeopathy **n = 445	**Conventional **n = 462	***p*-value, if < 0.05**

Male (%)	24.0	32.3	= 0.0064^b^
Female (%)	76.0	67.7	
Age	37.1 ± 12.5	39.6 ± 13.9	= 0.0124^a^
BMI	24.3 ± 4.8	25.0 ± 4.5	= 0.0031^a^
Smoking (%)	16.2	22.3	

Full-set analysis values are either expressed as % of total or as mean ± SD, ^a^Wilcoxon rank-sum test, ^b^Fisher's exact test.

As shown in Table 2, cough was the most frequently reported chief complaint in children, followed by sore throat and ear pain. In adults sore throat was the most frequent, followed by cough and runny nose. The overall distribution of the five chief complaints in children was comparable in both treatment groups, but differed significantly in adults (*p *= 0.0026, Chi-square test). The mean severity score differed significantly at baseline for 2 out of 5 chief complaints, both in children and adults (Table 2).

**Table 2:** Distribution and severity score of chief complaints at Day 0

**Children**	**Homeopathy **n = 407		**Conventional **n = 252		
**Chief complaint**	**(%)**	**Severity score**	**(%)**	**Severity score**	***p*-value**^**a**^** if < 0.05**
Runny nose	9.8	1.1 ± 0.5	15.5	1.9 ± 0.7	= 0.0001
Sore throat	24.6	1.7 ± 0.6	23.0	1.6 ± 0.6	
Ear pain	23.1	1.4 ± 0.6	21.0	1.0 ± 0.5	= 0.0002
Sinus pain	2.0	1.6 ± 0.4	3.6	1.7 ± 0.6	
Cough	40.5	0.9 ± 0.5	36.9	1.1 ± 0.6	

**Adults**	**Homeopathy **n = 445		**Conventional **n = 462		
**Chief complaint**	**%**	**Severity score**	**%**	**Severity score**	***p*-value**^**a**^** if < 0.05**

Runny nose	15.1	1.5 ± 0.8	14.7	1.9 ± 0.7	= 0.0005
Sore throat	43.4	1.6 ± 0.7	32.3	1.5 ± 0.6	
Ear pain	3.4	1.0 ± 0.3	5.4	1.3 ± 0.5	
Sinus pain	8.3	1.5 ± 0.6	13.4	1.5 ± 0.6	
Cough	29.9	1.0 ± 0.5	34.2	1.3 ± 0.5	= 0.0002

Full-set analysis values are either expressed as % of total or as mean ± SD. ^a^Wilcoxon rank-sum test, indicating the differences between severity scores (from 0 – not present to 4 – very severe) in the homeopathy and conventional group.

With regard to the diagnosis of the chief complaints, in children otitis media was most frequently diagnosed (H: 18.9%; C: 13.5%) followed by bronchitis (H: 16.7%; C: 10.7%) and laryngitis (H: 12.3%; C: 12.7%). In adults, pharyngitis (H: 23.1%; C: 14.7%), bronchitis (H: 11.5%; C: 17.1%) and tonsillitis (H: 13.9%; C: 8.9%) were most frequently diagnosed. In adults, no significant differences were observed with respect to concomitant medical problems (H: 34.2%; C: 36.6%) or concomitant medication (H: 20.7%; C: 20.1%). In the homeopathic group 21.6% of the children had concomitant medical problems versus 13.5% in conventional group (*p *= 0.0098; Fisher's exact test). The proportion of children receiving concomitant medication was higher in the homeopathic group (9.1%) than in the conventional group (6.7%) as well but did not reach a statistical significant level (*p *= 0.3098; Fisher's exact test).

### Medication

A total of 62 different homeopathic remedies were prescribed primarily on an individual basis. The top 10 (Table 3) of the most frequently prescribed homeopathic remedies included typical 'acute' remedies and accounted for about 60% of the prescriptions. In the conventional group 190 different medications were prescribed. Most of them were antibiotics followed by nasal preparations and analgesics (Table 3).

**Table 3:** The most frequently prescribed medications

**Children**		**Adults**	
**Homeopathic treatment **n = 407	**%**	**Homeopathic treatment **n = 445	**%**
1. Belladonna	13.3	1. Hepar sulphuris	9.7
2. Pulsatilla	10.6	2. Belladonna	8.3
3. Hepar sulphuris	6.6	3. Bryonia alba	7.2
4. Mercurius solubilis	6.4	4. Lycopodium clavatum	7.2
5. Phosphorus	4.9	5. Kalium bichromicum	5.8
6. Bryonia alba	3.7	6. Mercurius solubilis	4.9
7. Calcarea carbonica	3.7	7. Allium cepa	4.5
8. Lycopodium clavatum	3.7	8. Phosphorus	3.4
9. Sulphur	3.7	9. Causticum	3.1
10. Phytolacca decandra	3.4	10. Gelsemium sempervirens	2.7

**Conventional treatment **n = 252	**%**	**Conventional treatment **n = 462	**%**

1. Antibacterials	28.2	1. Antibacterials	39.4
2. Nasal preparations	22.6	2. Nasal preparations	15.2
3. Analgesics	12.7	3. Analgesics	9.5
4. Stomatological preparations	8.7	4. Cough/cold preparations	8.7
5. Anti-asthmatics	5.6	5. Stomatological preparations	5.2

### Treatment outcome

The primary outcome criterion, defined as the percentage of patients with complete recovery or major improvement after 14 days, was first calculated for the total patient population. The one-sided test of the full-set analysis showed non-inferiority of homeopathic in comparison with conventional treatment (H: 86.9%; C: 86.0%; *p *= 0.0003). These results were confirmed by the analysis on the per-protocol set (including all patients with data at day 14) since similar response rates were obtained in both treatment groups (H: 87.7%; C: 86.9%; *p *= 0.0019).

**Figure 2:**
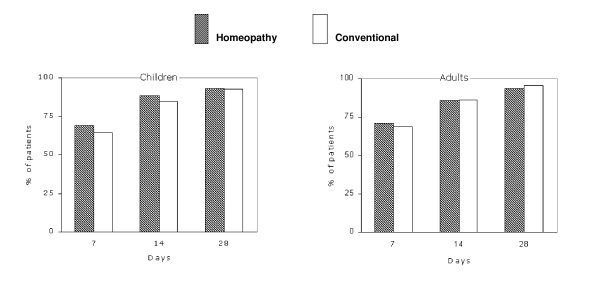
**Response rates after 7, 14 and 28 days of treatment**. Response rates (% of patients with complete recovery or major improvement) at 7, 14 and 28 days after treatment in children and adults. Full-set analysis with last observation carried forward (LOCF) at day 14 and 28. Children n = 659 (homeopathy, 407; conventional, 252) and adults n = 907 (homeopathy, 445; conventional, 462).

The response rates at various time points in children and adults are shown in Figure 2. The primary outcome criterion (response rate at day 14) in children was 88.5% after homeopathic and 84.5% after conventional treatment. In addition, response rates after 7 days (H: 68.8%; C: 64.3%) and 28 days (H: 93.1%; C: 92.5%) did not differ between both treatment groups either. In adults, the response rates after 7 days (H: 71.2%; C: 68.8%), 14 days (H: 85.6%; C: 86.6%, LOCF) and 28 days (H: 93.9%; C: 95.9%; LOCF) of treatment were not significantly different as well.

**Figure 3:**
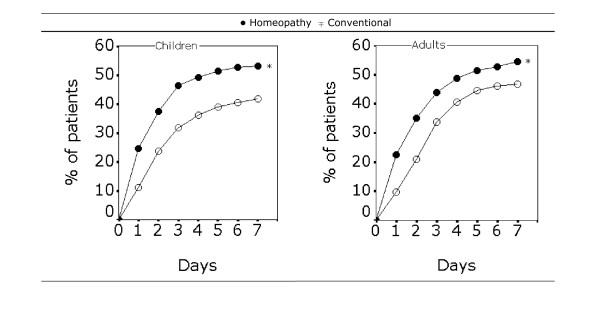
**Onset of improvement within the first week**. Onset of improvement within the first week of treatment (cumulative percentages of patients that experienced their first improvement). Children n = 659 (homeopathy, 407; conventional, 252) and adults n = 907 (homeopathy, 445; conventional, 462). Full-set analysis values with * *p *= 0.0448 for children and * *p *= 0.0001 for adults, using the Chi-square test on data points of the whole curve.

Since the majority of patients (> 84%) were fully recovered or major improved after 14 days of treatment, it was of relevance to look at outcome differences within the first 7 days. As shown in Figure 3, the percentage of children experiencing a first improvement at different time points within the first week of treatment was significantly higher in the homeopathy group compared to the conventional group (*p *= 0.0488). For adults, a similar significant difference in favour of homeopathy (*p *= 0.0001) was observed.

**Figure 4:**
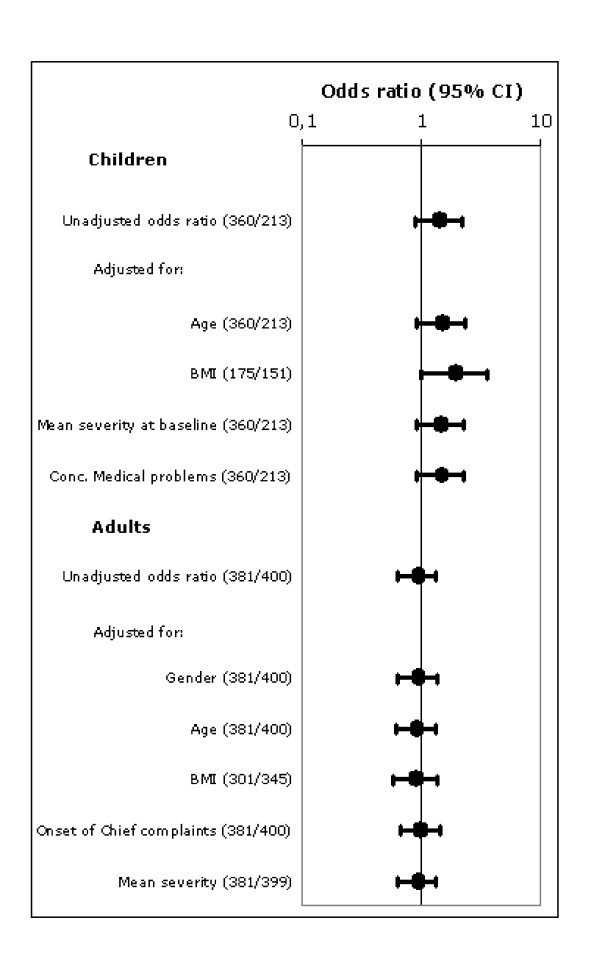
**Main outcome measure – corrections for baseline**. Main outcome measure: response to treatment (complete recovery or major improvement) of full-set analysis data at day 14, unadjusted odds ratio's and adjusted odds ratio's for baseline differences with 95% confidence intervals. Between brackets: the number of responders in the homeopathy group and conventional group, respectively. Odds ratio above 1 indicates a better outcome upon homeopathic treatment.

Additional analysis on the primary outcome criterion in order to correct for demographic differences at baseline was carried out (Figure 4). The unadjusted odds ratio (OR) of the primary outcome criterion was 1.40 (0.89–2.22) for children and 0.92 (0.63–1.34) for adults. In the subgroup of children, adjustments for age, mean severity and concomitant medical problems had little effect on the OR. The unadjusted OR for the Body-Mass-Index was 1.92 (1.03–3.60) and the only one showing a significant difference in favour of homeopathy. Adjustment for BMI differences between both treatment groups at baseline minimally reduced the OR to 1.89 (1.00–3.57). In adults, individual adjustments for all variables had little to no effect on the OR of the primary outcome criterion (Figure 4).

Another outcome measure was the occurrence of adverse drug reactions. The percentage of children experiencing a suspected adverse drug reaction was not significantly different in both groups (H: 2.0%; C: 2.4%, *p *= 0.7838, Fisher's exact test). In adults, the number of suspected adverse drug reactions was significantly higher after conventional than after homeopathic treatment (C: 7.6%; H: 3.1%; *p *= 0.0032, Fisher's exact test). Both in children and adults, the suspected adverse drug reactions occurred predominantly in the body as a whole (upon homeopathic treatment) or in the gastro-intestinal system (upon conventional treatment).

In addition, patients' satisfaction with treatment and healthcare provider was evaluated. Almost all patients in both treatment groups were either satisfied or very satisfied with the treatment after 28 days (children: 95% H; 93% C, adults: 91% H; 95% C). A very high percentage of children (H: 98%; C: 95%) and adults (H: 97%; C: 97%) were either satisfied or very satisfied with the healthcare provider.

## Discussion

The overall outcome of the first phase of the IIPCOS study [12] is confirmed in the present study on a larger group of patients and a greater number of medical practices, showing that homeopathic treatment is not inferior to conventional treatment for the treatment of acute respiratory and ear complaints. In IIPCOS-1 the response rate of homeopathically treated patients was with 82.6% significantly higher than in the conventional group. In IIPCOS-2 the response to homeopathic treatment was with 86.9% even higher, confirming the good effectiveness. However, no difference was observed between both treatment groups. This is due to a much higher response rate in the conventional group in IIPCOS-2 of 86.0% compared to 68% in IIPCOS-1. One difference between both studies is that in IIPCOS-2, only patients from Europe were analysed since those recruited at practices from the USA were excluded due to protocol deviations. In IIPCOS-1, the majority of patients included had their residence in the USA. However, despite these differences, the overall conclusion from both studies can be drawn that homeopathy is not inferior to conventional therapy. Due to the study design, the findings of IIPCOS-1 and IIPCOS-2 do not provide firm data on the comparative efficacy of homeopathic and conventional treatment in acute diseases but rather underline the potential value of homeopathy in every day clinical practice. Both studies reflect the situation in every day homeopathic practice in an international setting with average patients receiving the usual treatment of a homeopathic doctor. Furthermore, patients were recruited on the basis of chief complaints and related symptoms, rather than on the clinical diagnoses. This symptomatic approach coincides with the homeopathic nature of prescription by treating each patient individually, based on specific key symptoms and patient characteristics.

In IIPCOS-2, differences for various demographic parameters and symptom-related variables were found between both groups. Thereby the profile of typical patients seeking homeopathic therapy was confirmed [13,14], i.e. they were more likely to be women, younger of age, less likely to smoke and to have a lower BMI. The severity of symptoms at baseline was significantly different between treatment groups as well. However the differences were small and their clinical relevance is doubtful. Indeed regression analysis had little effect on the primary outcome criterion, showing that treatment effects were only minimally affected by selection bias. Based on the unadjusted and adjusted odds ratios of the primary outcome criterion it appears that homeopathic treatment, in comparison to conventional treatment, is more beneficial for children than adults. This observation is in accordance with previous studies in which the improvements after homeopathic treatment were greater in children than in adults [11,13].

Another possible source of bias is that the outcome criteria were assessed by the patients themselves. Since it was not possible to blind patients for their treatment, potential reporting bias from patient's expectations may have influenced the outcome. On the other hand, the patients' reports were collected by independent external study collaborators in order to minimize the influence of the patient's relationship with their physician on the treatment outcome. Although blinding of the external study coordinators was foreseen in the protocol, it cannot be ruled out that they received information from the patient revealing the nature of their medication. Therefore, blinding may not have been guaranteed in each case. Furthermore, it should be noted that at mixed centres, the choice of treatment was made by the physicians and/or following the patients' preference. The treatment decision may have been influenced by the kind or severity of the symptoms or the motivation and expectations of the patient.

Since acute respiratory and ear complaints are self-limiting conditions, it can be argued that the chosen primary outcome criterion after 14 days of treatment is not sufficiently sensitive. Patients experiencing these acute complaints may have undergone spontaneous recovery within 1 to 2 weeks. However, this outcome parameter was taken to confirm and reproduce the results of IIPCOS-1 by using a similar study design. Therefore other outcomes criteria such as the response rate after 7 days of treatment have to be considered more carefully. Moreover, the findings that the percentage of patients experiencing a first improvement within the first week was higher at all time points in the homeopathy group than in the conventional group, are at least supportive of the 14 days finding that homeopathy is not inferior to conventional medicine.

Other observational studies on the comparability of homeopathic treatment and conventional treatment of upper respiratory tract infections (URTI) have shown positive outcomes for homeopathy [15,16]. Recently, the value of homeopathic treatment for the prevention of URTIs has been demonstrated in a controlled clinical trial [17]. The consistent findings in IIPCOS-1 and IIPCOS-2 further contribute to the evidence that homeopathic treatment plays a beneficial role in the primary care of patients. Furthermore, the good tolerability of homeopathic treatment of acute respiratory and ear complaints was confirmed by the low number of patients that experienced adverse drug reactions.

The major limitation of the present study is that patients were not assigned randomly to their treatment group. The majority of patients in the homeopathic group had a strong treatment preference and consequently, they were not willing to be randomized. A similar reluctance towards randomisation has also been reported elsewhere for patients seeking anthroposophic therapy [18]. These results reveal a substantial limitation to the suitability of performing large randomized controlled trials on the efficacy of homeopathy in such a primary care setting.

## Conclusion

This comparative cohort study, involving more than 1,500 patients in primary care practices of at least 6 different European countries, demonstrates that homeopathic treatment for acute respiratory and ear complaints was not inferior to conventional treatment. Although no firm conclusions can be drawn about the efficacy of homeopathic treatment, these results certainly contribute to the growing evidence that homeopathy is a safe and beneficial treatment strategy for acute diseases in primary care settings.

## Competing interests

MJ is an employee and MHe † was an employee of the HomInt organisation. All other authors have no financial or non-financial competing interest related to the content of the manuscript.

## Authors' contributions

International Integrative Primary Care Outcomes Study 2 (IIPCOS-2) collaborators: MH, DR and MHe planned and directed the study. SB, GL, GJ and AT were responsible for data collection. MJ drafted the manuscript. MF performed the statistical analysis. All authors read and approved the final version of the manuscript.

## Pre-publication history

The pre-publication history for this paper can be accessed here:


